# Clinical trials in older patients with cancer – typical challenges, possible solutions, and a paradigm of study design in breast cancer

**DOI:** 10.2340/1651-226X.2023.40365

**Published:** 2024-06-17

**Authors:** Peeter Karihtala, Aglaia Schiza, Elena Fountzilas, Jürgen Geisler, Icro Meattini, Emanuela Risi, Laura Biganzoli, Antonios Valachis

**Affiliations:** a Department of Oncology, Helsinki University Hospital Comprehensive Cancer Center and University of Helsinki, Helsinki, Finland; b Department of Oncology, Uppsala University Hospital and department of Immunology, Genetics and Pathology Uppsala University, Uppsala, Sweden; c Department of Medical Oncology, St. Luke’s Clinic, Thessaloniki, Greece; d Institute of Clinical Medicine, Faculty of Medicine, University of Oslo, Oslo, Norway & Akershus University Hospital, Department of Oncology, Lørenskog, Norway; e Department of Experimental and Clinical Biomedical Sciences “M. Serio”, University of Florence, Florence, Italy; f Radiation Oncology and Breast Unit, Oncology Department, Careggi University Hospital, Florence, Italy; g Department of Oncology, Hospital of Prato, Azienda USL Toscana Centro, Italy; h Department of Oncology, Faculty of Medicine and Health, Örebro University, Örebro, Sweden

**Keywords:** Barriers to recruitment, breast cancer, clinical trials, elderly, older patients

## Abstract

**Background and purpose:**

While the prevalence of older breast cancer patients is rapidly increasing, these patients are greatly underrepresented in clinical trials. We discuss barriers to recruitment of older patients to clinical trials and propose solutions on how to mitigate these challenges and design optimal clinical trials through the paradigm of IMPORTANT trial.

**Patients and methods:**

This is a narrative review of the current literature evaluating barriers to including older breast cancer patients in clinical trials and how mitigating strategies can be implemented in a pragmatic clinical trial.

**Results:**

The recognized barriers can be roughly divided into trial design-related (e.g. the adoption of strict inclusion criteria, the lack of pre-specified age-specific analysis), patient-related (e.g. lack of knowledge, valuation of the quality-of-life instead of survival, transportation issues), or physician-related (e.g. concern for toxicity). Several strategies to mitigate barriers have been identified and should be considered when designing a clinical trial dedicated to older patients with cancer. The pragmatic, de-centralized IMPORTANT trial focusing on dose optimization of CDK4/6 -inhibitors in older breast cancer patients is a paradigm of a study design where different mitigating strategies have been adopted.

**Interpretation:**

Because of the existing barriers, older adults in clinical trials are considerably healthier than the average older patients treated in clinical practice. Thus, the study results cannot be generalized to the older population seen in daily clinical practice. Broader inclusion/exclusion criteria, offering telehealth visits, and inclusion of patient-reported, instead of physician-reported outcomes may increase older patient participation in clinical trials.

## Introduction

Over a third of all new invasive breast cancer cases are diagnosed in patients aged 70 years and older in Western societies, and the median age is constantly increasing [1–3]. There is also convincing data that older patients with breast cancer have shorter survival compared to younger patients, possibly due to cancer diagnosis at later stages and administration of less intensive treatments [4–6].

Despite the increasing prevalence of the older breast cancer population, these patients are substantially underrepresented in clinical trials [7, 8]. In an analysis of systemic therapy trials in breast cancer patients published between 1985 and 2012, only 7% and 15% of patients aged 70 years and older participated in adjuvant and metastatic trials, respectively [9]. The enrollment of older breast cancer patients with metastatic disease was also decreasing over time. With increasing age, the underrepresentation is even more prominent [10].

In addition to their increasing breast cancer prevalence, it is essential to include the older population in clinical trials since their pharmacokinetics and pharmacodynamics are likely to vary due to naturally occurring organ impairments and interactions with other drugs [11]. Although age alone does not reflect an intolerability to oncological systemic therapies, older patients still undergo arbitrary upfront dose reductions in clinical practice [12]. Despite their well-recognized benefits, geriatric assessments to determine biological frailty and social or psychological challenges are still rarely used in clinical practice or in oncological studies [1, 13–16]. The use of comprehensive geriatric assessment (CGA) in oncological phase I, II and III studies has increased since the beginning of 2000s; however, CGA was still used only in 11% of the clinical trials performed between 2011 and 2014 [17].

In this narrative review, we describe challenges in designing and conducting clinical trials for older patients, provide strategies to mitigate these obstacles and discuss how novel trial designs could be conducted to meet these challenges. To illustrate the adoption of different mitigating strategies in study design and conduct, we use the paradigm of IMPORTANT trial that is dedicated to older patients with advanced hormone-receptor (HR)-positive/HER2-negative breast cancer planned to be treated with cyclin-dependent kinase 4/6 (CDK4/6) -inhibitors and endocrine therapy in the first-line setting. Although our primary focus is on breast cancer, most of the interpretations apply to all malignancies.

## Challenges in designing and conducting clinical trials for older cancer patients

**Figure 1:**
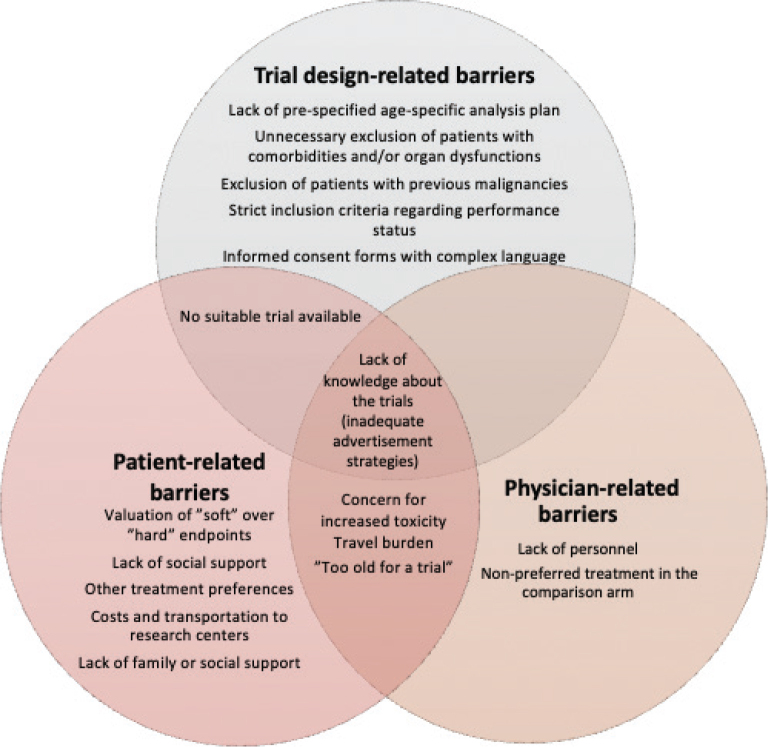
Overview of identified barriers to older patients’ participation in clinical oncological trials. PS = performance score.

Several studies have investigated barriers that lie behind the under-representation of older cancer patients in clinical trials as a first step to design and adopt mitigating strategies [18–24]. These barriers can be roughly divided into trial design-related, patient-related, or physician-related barriers (Figure 1).

### Trial design-related barriers

A major barrier to include older patients in clinical trials is the adoption of strict inclusion and exclusion criteria that leads to exclusion for the vast majority of older patients. Although increased age *per se* is rarely an exclusion criterion in breast cancer trials, there are several indirect reasons leading to the exclusion of a substantial proportion of older patients from clinical trials. One of the main indirect exclusion criterion for older adults is the restriction to include only patients with Eastern Cooperative Oncology Group performance status (PS) of 0 to 1. In contrast with the general population, up to 96% of the participants in phase III cancer trials were reported to have PS of 0 to 1 [25]. The proportion of older cancer patients was reported to be 22% lower in the trials excluding patients with mild or moderate functional status impairment compared to trials not excluding these patients [26]. In addition, the subjectivity of the PS scoring remains an unsolved issue and poses additional challenges in including older cancer patients in clinical trials. Other indirect exclusion criteria of older patients comprise comorbidities and/or organ dysfunction [27–29]. Patients with previous malignancies have been excluded from up to 90% of clinical oncological trials [26]. This may significantly decrease the participation of older patients who have a higher risk of reporting a previous cancer diagnosis. Still, including these patients in clinical trials would not affect outcomes, especially in the early-phase trials with primary endpoints of toxicity. Consequently, older adults in trials have fewer functional impairments and fewer comorbidities than the average older patient treated in clinical practice and, therefore, the results cannot be inferred to the general older population.

Another barrier related to trial design is the lack of a pre-specified age-specific analysis plan. In an analysis of 159 clinical oncological trials, only 39.9% reported effectiveness by age, while 8.9% reported adverse events by age [30]. For instance, *post hoc* data showed that CDK4/6 inhibitors have similar efficacy, but higher rates of toxicity and dose modifications in patients older than 75 years than in the younger clinical study participants [31]. Other frequently recognized, trial-related barriers include the presence of lengthy informed consent forms with complex language, along with the adoption of communication and advertisement strategies for the trial that do not cover the preferences of an older population [18, 21, 32].

### Patient-related barriers

One of the most common patient-related limitations of participation in clinical oncological studies, highlighted in the older population, is the lack of knowledge about possible clinical trials [7, 9, 20, 33]. While younger patients are increasingly seeking information about potential clinical trials from the internet, older adults are less likely to have access to electronic literacy [34]. The expectations of younger and older participants in clinical trials may also differ. While most oncological phase II-III studies commonly use primary endpoints as response rate, disease progression, or improvement in overall survival, older cancer patients frequently prioritize maintenance of quality of life and function over improved survival [35]. Providing trials that would emphasize patient-reported, instead of physician-reported outcomes and pragmatic de-escalation studies with non-inferiority hypotheses, could be very beneficial for this population and their treating physicians.

Although some studies have reported that older patients are more likely to believe that being on a clinical trial would provide better treatment and follow-up care, there are also concerns among older patients about the possibility that investigational drugs might lead to increased toxicity and worsened quality of life [18, 36]. Other commonly reported patient-related barriers include having other treatment preferences, a lack of social support, and perceptions of family being against trial participation [8, 18]. Potential costs and issues related to transportation to university centers have been also mentioned as frequent causes precluding older patients from participating in clinical trials [18, 19, 21].

### Physician-related barriers

There are also several physician-related barriers that can influence the possibility of trial participation among older patients. Patient age itself has been recognized as a physician-related barrier leading to reduced recruitment into clinical trials in various studies and in a recent systematic review [8, 21, 22, 29, 32]. By far, the most common reason for not offering a trial participation specifically for older adults is not having an applicable trial, in up to 75% of cases [37]. Interestingly, older adults are still just as likely to agree to participate in a clinical trial compared to younger women if they were offered enrollment [13]. Another physician-related reason not to offer or enroll older patients into clinical trials seems to be a concern for toxicity [18, 29, 36, 38], while less common reasons include discomfort with the randomization and non-preferred treatment in the comparison arm of the trial [8]. Time burden and a lack of personnel emerged as physician-related barriers that have been recognized in the literature as well [39]. Finally, trials in general demand many additional appointments and investigations that may be difficult to reach for older patients without permanent taxi transport and/or accompanying family members [9, 18, 19].

As a paradigm on under-representation of older patients with cancer on pivotal, practice-changing randomized trials, Table 1 presents the landscape of older patients with metastatic breast cancer included in pivotal trials that have led to European Medical Agency’s approval for clinical use in Europe during the period 2016–2023.

**Table 1:** Landscape of older patients with metastatic breast cancer included in pivotal trials with treatment strategies that are approved for clinical use during the period 2016–2023

Trial (reference)	Experimental treatment	Total number of patients	Age ≥ 65 years N (%)	Age ≥ 70 years N (%)	Dedicated analysis for older patients
***Hormone receptor positive / HER2-negative (including HER2-low) breast cancer***					
Pooled analysis of PALOMA -1, -2-, -3 [40]	Palbociclib + aromatase inhibitors Palbociclib + fulvestrant	1352	304 (22.5)	NR (83 patients ≥ 75 years)	Posthoc subgroup analysis
MONALEESA-2 [41]	Ribociclib + aromatase inhibitors	668	295 (44.1)	NR (upper age range 92 years)	Predefined subgroup analysis (age threshold 65 years)
MONALEESA-3 [42]	Ribociclib + fulvestrant	726	339 (46.7)	NR (upper age range 89 years)	Predefined subgroup analysis (age threshold 65 years)
SOLAR-1 [43]	Alpelisib + fulvestrant	572	NR (median age 62–64 years)	NR (upper age range 92 years)	No separate analysis
Pooled analysis of MONARCH-2 and -3 [44]	Abemaciclib + aromatase inhibitors Abemaciclib + fulvestrant	1152	464 (40.3)	NR (133 patients ≥ 75 years)	Posthoc subgroup analysis
DESTINY-BREAST04 [45]	Trastuzumab-deruxtecan (HER2-low)	557	114 (20.5)	NR	No separate analysis
***HER2-positive breast cancer***					
DESTINY-BREAST03 [46]	Trastuzumab-deruxtecan (2^nd^ line)	524	NR (median age 54 years)	NR (upper IQR 63 years)	No separate analysis
DESTINY-BREAST02 [47]	Trastuzumab-deruxtecan (3^rd^ or later line)	608	NR (median age 54 years)	NR (upper IQR 63 years)	No separate analysis
HER2CLIMB [48]	Tucatinib + trastuzumab + capecitabine	612	116 (19.0)	NR (upper age range 79 years)	Predefined subgroup analysis (age threshold 65 years)
***Triple-negative breast cancer***					
IMPassion130 [49]	Atezolizumab + nab-paclitaxel	902	219 (24.3)	NR (upper IQR 65 years)	Predefined subgroup analysis (age threshold 65 years)
KEYNOTE-355 [50]	Pembrolizumab + chemotherapy	847	180 (21.3)	NR (upper IQR 63 years)	Predefined subgroup analysis (age threshold 65 years)
ASCENT [51]	Sacituzumab govitecan	468	90 (19.2)	NR (upper age range 82 years old)	Predefined subgroup analysis (age threshold 65 years)
***Germline BRCA1/2 mutation***					
OlympiAD [52]	Olaparib	302	NR (median age 44 years)	NR (upper age range 76 years old)	No separate analysis
EMBRACA [53]	Talazoparib	431	NR 42% ≥ 50 years old)	NR (upper age range 88 years old)	No separate analysis

NR: not reported; IQR: interquartile range.

## Strategies to mitigate challenges

Different strategies need to be employed to increase the recruitment and retention of older patients in clinical breast cancer trials. Importantly, patient awareness and education need to be focused on the significance of participation in clinical trials, on the potential clinical benefits a patient might derive from an innovative treatment, while addressing misconceptions and fears patients might have about adverse events or receiving treatment with a placebo instead of an active regimen [20, 54]. This information needs to be provided to the older patient in a protected environment, providing sufficient time for comprehension, utilizing visual aids and plain words and concepts about the procedures of the clinical trial, in a process that is tailored to the needs of each older patient. Companion care from a family member or another caregiver is of critical significance to ensure patient emotional and physical support throughout informed consent and other trial procedures.

Patient commitment can also be increased by facilitating their participation in the trial. For instance, accommodating older patient transportation to the trial site with dedicated services and/or reimbursing travel expenses, would be of great importance, especially for underserved populations or patients living in remote places. Alternatively, shorter appointments, decreased number of visits, or ideally, substitution of patient visits by telehealth visits would accommodate the special needs and difficulties of older patients while ensuring their safety, thus motivating trial participation and protocol adherence. Current data have demonstrated that the use of digital tools, such as mobile apps and wearable devices, based on user-friendly technologies facilitate data collection, participant communication, and close monitoring for adverse events [55, 56].

The procedures that may burden an older participant, such as repeated biopsies, multiple tests, other invasive procedures and complex scheduling should be minimized to the greatest extent possible. Finally, all trial procedures, including patient follow-up appointments, need to be performed and monitored by a multidisciplinary healthcare team, one that can identify and address the special needs of an older patient. By implementing these strategies, older patient recruitment and retention can be enhanced, thus providing innovative treatment options to older patients and important clinical information to the medical community on efficacy and toxicity data on the older population.

## Adopting mitigating strategies to trial design and conduct – the paradigm of IMPORTANT trial

Recognizing the challenges in designing and conducting clinical trials for older cancer patients and taking into account the growing body of evidence on barriers to the inclusion of older cancer patients in trials, the IMPORTANT trial tried to adopt several strategies to mitigate these barriers. IMPORTANT trial is a pragmatic randomized controlled trial investigating whether a lower initial dose of CDK4/6 -inhibitors combined with endocrine therapy in older patients with advanced HR-positive/HER2-negative breast cancer categorized as vulnerable/frail according to CGA is comparable to a full dose.

To avoid trial design-related barriers, IMPORTANT study has been designed as a dedicated clinical trial for older breast cancer patients. Broad eligibility criteria have been adopted to achieve a study cohort that will be representative of patients seen in clinical practice (including men with breast cancer which is an overlooked patient subgroup in all pivotal clinical trials on CDK4/6 -inhibitors). As an additional effort to broaden the study inclusion, IMPORTANT study expands the enrollment to community practices through satellite clinical sites to enable a broader patient enrollment. Measuring relevant endpoints for this patient group and not only efficacy and toxicity data that might not always be relevant in a geriatric population is another crucial aspect when designing clinical trials dedicated to older cancer patients [57]. IMPORTANT study has, therefore, chosen to include composite endpoints such as overall treatment utility, as well as patient-reported quality-of-life measures, and aging-related measures as endpoints of interest, whereas the composite endpoint time-to-treatment failure is chosen as the primary endpoint.

To further tailor the study design for older cancer patients, IMPORTANT study incorporates a CGA at baseline that will be a part of the decision-making process enabling a more individualized treatment strategy, thus empowering shared decision making. Incorporating geriatric assessment tools in treatment decision-making for older cancer patients is recommended by international guidelines, but hardly implemented in clinical practice [1, 15].

Regarding patient-related barriers, IMPORTANT study has adopted decentralized approaches (capture data on geriatric assessment and quality-of-life through easy-to-use electronic platforms, use of telemedicine for toxicity evaluation to minimize the in-hospital visits) that combine participant-centered design with innovative technologies to reduce the need for physical in-person interaction between participants and researchers. Such de-centralised, pragmatic approaches have been shown to be able to improve patients’ willingness to enroll in clinical trials, including older cancer patients as well as reduce the burden related to transportation and costs [58, 59]. Decentralized approaches might also have an impact on caregivers’ positive view of clinical trial participation [60].

Regarding physician-related barriers, IMPORTANT study adopted a pragmatic design in terms of both the treatment strategies, where standard-of-care treatment with CDK4/6 -inhibitors and endocrine therapy is offered to all study participants and follow-up strategies that resemble the current follow-up strategy in clinical practice without unnecessary blood tests or radiological examinations. These two aspects can overcome barriers related to physicians’ concerns about additive toxicity due to investigational drugs or potential preference for other treatment (the patients would receive the same treatment outside of the study) and also barriers associated with a lack of personnel and time in clinical practice (no study-related visits or additional examinations).

## Conclusions and future directions

As the breast cancer population ages, it is essential for older patients, caregivers and also drug developers to include these patients in clinical trials to produce evidence that can be implemented into clinical practice for this specific population. This is of particular importance considering the risks for arbitrary dose reduction that might impact treatment efficacy when the results from non-representative trials are generalized to older patients in clinical practice [12]. Recognizing barriers related to the inclusion of older cancer patients in clinical trials is the first step in designing and implementing strategies to mitigate these barriers. The most promising strategy to mitigate these barriers could be the design and conduct of clinical trials dedicated specifically to older cancer patients.

## Data Availability

As a review article, there is no original data in this manuscript.
